# Angiogenin mutations in Hungarian patients with amyotrophic lateral sclerosis: Clinical, genetic, computational, and functional analyses

**DOI:** 10.1002/brb3.1293

**Published:** 2019-04-25

**Authors:** Kornélia Tripolszki, Judit Danis, Aditya K. Padhi, James Gomes, Renáta Bozó, Zsófia F. Nagy, Dóra Nagy, Péter Klivényi, József I. Engelhardt, Márta Széll

**Affiliations:** ^1^ Department of Medical Genetics University of Szeged Szeged Hungary; ^2^ MTA‐SZTE Dermatological Research Group Szeged Hungary; ^3^ Kusuma School of Biological Sciences Indian Institute of Technology Delhi New Delhi India; ^4^ Department of Dermatology and Allergology University of Szeged Szeged Hungary; ^5^ Department of Neurology University of Szeged Szeged Hungary; ^6^ Present address: Laboratory for Structural Bioinformatics Field for Structural Molecular Biology Centre for Biosystems Dynamics Research RIKEN Yokohama Japan

**Keywords:** amyotrophic lateral sclerosis, angiogenin, molecular dynamics, mutation screening, nuclear translocation, ribonucleolytic activity

## Abstract

**Introduction:**

Mutations in the *angiogenin* (*ANG*) gene are known to be associated with both familial and sporadic amyotrophic lateral sclerosis (ALS). The majority of disease‐causing mutations of *ANG* result in loss of either ribonucleolytic activity, nuclear translocation activity or both.

**Methods:**

We sequenced *ANG* gene from a total of 136 sporadic ALS patients and 112 healthy controls of Hungarian origin. To elucidate the role of the R33W mutation in the disease mechanism, computational, and functional analyses were performed.

**Results:**

Mutation screening revealed a mutation located in the signal peptide (M‐24I) and two mutations that affect the mature protein (R33W, V103I). The R33W mutation, which has not been previously detected in ALS patients, affects the key amino acid of the nuclear translocation signal of the ANG protein. Molecular dynamics simulations suggested that the R33W mutation results in partial loss of ribonucleolytic activity and reduced nuclear translocation activity. The ribonucleolytic assay and nuclear translocation assay of the R33W ANG protein confirmed the molecular dynamics results.

**Conclusions:**

In the Hungarian ALS population, the observed frequency of *ANG* mutations was 2.9%, which is higher than previously reported for sporadic cohorts. The evidence from computational and functional analyses support the deleterious effect of the novel R33W variant detected in this study.

## 1 INTRODUCTION

Amyotrophic lateral sclerosis (ALS) is a fatal neurodegenerative disease characterized by the loss of motor neurons leading to paralysis and death 3–5 years after disease onset. Approximately 90%–95% of cases are sporadic with no family history (sALS), whereas the remaining 5%–10% is familial (fALS; Renton, Chiò, & Traynor, 2014); however, the clinical features of fALS and sALS are almost indistinguishable. In the past decades, more than 30 genes involved in the etiology of the disease have been identified (Amyotrophic Lateral Sclerosis Online Genetics Database, ALSoD; Abel, Powell, Andersen, & Al‐Chalabi, 2012). The prevalence of mutations of the two most frequently mutated genes, *SOD1* and *C9orf72* repeat expansion was previously reported for the Hungarian population (Tripolszki et al., 2017).

**Figure 1:**
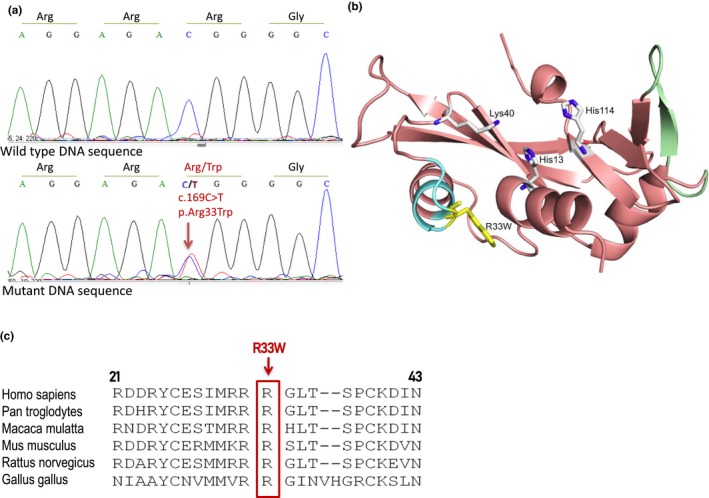
The R33W mutation identified in the *angiogenin* (*ANG*) gene in a Hungarian sporadic amyotrophic lateral sclerosis patient. (a) Electropherograms of wild‐type and mutant *ANG* sequences. (b) X‐ray structure of human ANG in cartoon representation showing its functional sites and R33W mutation. Cartoon representation of the structure of human ANG (Protein Data Bank: 1B1I) showing its functional sites: catalytic triad residues His13, Lys40, and His114 are shown as stick models; nuclear localization signal is shown in cyan; and receptor binding sites are shown in green. The R33W mutation is represented as stick model in yellow. (c) Alignment of ANG protein sequences from various species shows the conservation of the R33 residue

First reported in 2004, substantial evidence has accumulated for *angiogenin* (*ANG*) involvement in ALS (Greenway et al., 2004; Sheng & Xu, 2016). To date, approximately 30 different mutations are reported for *ANG* in the ALSoD (Abel et al., 2012). The *ANG* gene, located on chromosome 14q11.2, encodes ANG, a 123‐residue, 14.1‐kDa protein. The ANG protein belongs to the pancreatic ribonuclease superfamily (Strydom et al., 1985), and it plays an important role in rRNA biogenesis and cellular proliferation (Kishimoto, Liu, Tsuji, Olson, & Hu, 2005) in addition to its crucial role in inhibiting protein translation by cleaving transfer RNA (tRNA; Ivanov, Emara, Villen, Gygi, & Anderson, 2011). ANG is synthesized with a signal peptide of 24 amino acids that is cleaved to form the mature protein (Fett et al., 1985; Shapiro, Riordan, & Vallee, 1986). The mature protein contains three functional sites: a receptor binding region ^60^NKNGNPHREN^68^, the catalytic triad His13, Lys40, and His114, which is responsible for ribonuclease activity, and a nuclear localization signal sequence ^29^IMRRRGL^35^, which is responsible for translocation into the nucleus (Figure 1; Hallahan, Shapiro, Strydom, & Vallee, 1992; Leonidas et al., 1999; Moroianu & Riordan, 1994). It is known that ANG mediates neurovascularization and promotes neurite outgrowth during early embryonic development and that it protects against hypoxia‐induced motor neuron death (Kishimoto et al., 2005; Moroianu & Riordan, 1994; Sebastià et al., 2009). Mutations in the *ANG* gene cause loss of either ribonucleolytic activity or nuclear translocation activity or both (Wu et al., 2007).

The aim of this study was to evaluate the contribution of mutations in *ANG*, a major ALS gene, to the pathogenesis of the disease in Hungarian patients. The patients positive for *SOD1* or *C9orf72* repeat expansion identified in our previous study (Tripolszki et al., 2017) were not excluded, as there is evidence that ALS is oligogenic (van Blitterswijk et al., 2012). To explore the underlying mechanisms that result in loss of function of the detected R33W mutant protein, computational simulations, ribonucleolytic assays, and nuclear translocation assays were performed.

## 2 PATIENTS AND METHODS

### 2.1 Patients

The investigated sporadic patients (*n* = 136) were recruited from the Department of Neurology, University of Szeged, Szeged, Hungary. All patients fulfilled the revised El Escorial criteria for ALS (Ludolph et al., 2015). Among the patients, 54 were males and 82 were females (M/F ratio was 1:1.51), and the mean age of disease onset was 60.72 years (range 29–86 years). All patients were of Hungarian ancestry. The study was approved by the Internal Ethical Review Board of the University of Szeged. Written informed consent was obtained from all patients, and the study was conducted according to the Principles of the Declaration of Helsinki.

### 2.2 Methods

#### 2.2.1 Genetic analysis

Genomic DNA was isolated from frozen blood using DNeasy Blood and Tissue kit (QIAGEN, Gödöllő, Hungary). The coding region and the flanking introns of the *ANG* gene were amplified (primer sequences used were taken from Fernández‐Santiago et al., 2009). Direct sequencing of the PCR products was performed on an ABI 3100 sequencer and compared with the reference gene sequences (*ANG*, RefSeq NM_001145). To identify known variations, ALSoD (http://alsod.iop.kcl.ac.uk/, Abel et al., 2012), 1,000 Genomes Database (www.1000genomes.org/), dbSNP (http://www.ncbi.nlm.nih.gov/project/SNP), and gnomAD (Lek et al., 2016) databases were used. To predict the functional effects of mutations, the combination of seven variant prioritization tools available from the dbNSFP database v3.0 (MetaSVM, MetaLR, CADD, PROVEAN, SIFT, MutationTaster, MutationAssessor) were used (Liu, Wu, Li, & Boerwinkle, 2016). Amino acid numbering of ANG is according to the ALS Online Database (Abel et al., 2012), which has been used in the previous published reports on *ANG* mutations.

#### 2.2.2 System setup and all‐atom molecular dynamics simulations

The crystal structure of the human ANG protein (entry: 1B1I) was retrieved from the RCSB Protein Data Bank and used as the starting structure (Leonidas et al., 1999). The R33W variant identified in the Hungarian patient was modeled in silico by mutating Arg33 residue to Trp33, while keeping the secondary structure intact. The cofactor, CIT and crystallographic waters were removed from the structure and hydrogen atoms were added subsequently using the Xleap tool of AMBER 14 (Case et al., 2014). Modeled structures for the wild‐type and R33W proteins were then solvated in an octahedral box of TIP3P water with ~10 Å between the protein surface and the box boundary (Jorgensen, Chandrasekhar, Madura, Impey, & Klein, 1983). Each system was then electrostatically neutralized by the addition of Cl^−^ counter ions and simulations were carried out using the SANDER module of the AMBER 14 software package with ff14SB force field (Maier et al., 2015).

Generation of topology and parameter files, energy minimization, heating and temperature equilibration, production run, and MD trajectory analysis was carried out as described previously (Padhi, Banerjee, Gomes, & Banerjee, 2014; Padhi & Gomes, 2019; Padhi, Kumar, Vasaikar, Jayaram, & Gomes, 2012). All figures for representing ANG structures were generated using PyMOL and visual molecular dynamics (VMD). A VMD plug‐in, VolArea, was used to calculate the solvent‐accessible surface area (SASA) of nuclear localization signal residues ^31^RRR^33^ using a probe radis of 1.4 Å for evaluating the nuclear translocation activity (Humphrey, Dalke, & Schulten, 1996; Ribeiro, Tamames, Cerqueira, Fernandes, & Ramos, 2013).

#### 2.2.3 Solvent‐accessible surface area

The nuclear translocation activity of the R33W variant was examined by computing the SASA of nuclear localization signal residues ^31^RRR^33^. A VMD plug‐in, VolArea, was used to calculate the SASA of wild‐type and R33W using a probe radius of 1.4 Å (Humphrey et al., 1996; Ribeiro et al., 2013).

#### 2.2.4 Graphics and figure preparation

All figures for representing ANG structures were generated using PyMOL (http://www.pymol.org) and VMD. Hydrogen bond interactions were visualized using Cytoscape (Shannon et al., 2003).

#### 2.2.5 Ribonucleolytic activity assay

The ribonucleolytic activity of the wild‐type and mutant ANG protein was determined by measuring their activity toward yeast tRNA (Shapiro et al., 1986). The 30 μl assay mixtures contained 0.05–0.4 mg/ml wild‐type or R33W mutant ANG (14.3 kDa) with >95% purity (Novoprotein Scientific, Summit, NJ), 30 mM 4‐(2‐hydroxyethyl)‐1‐piperazineethane‐sulfonic acid, pH 6.8 (Sigma), 30 mM sodium chloride (Sigma), 3 μg ultrapure RNAse‐free bovine serum albumin (BSA, Thermo Fisher), and 60 μg yeast tRNA (Sigma). After 2 hr of incubation at 37°C, 70 μl of 3.4% ice‐cold perchloric acid (Sigma) was added to terminate the reaction. After vortexing, the mixtures were incubated on ice for 15 min, and centrifuged at 15,000 *g* for 10 min at 4°C. Supernatant absorbance was measured at 260 nm with a MaestroNano spectrophotometer. Data were collected from three independent experiments.

#### 2.2.6 Nuclear translocation assay

Human umbilical vein endothelial cells (HUVEC; Cell Applications Inc., San Diego, CA) were grown in Endothelial Cell Growth Medium (Cell Applications) in a humidified atmosphere with 5% CO_2_ at 37°C. Cells were seeded at 5 × 10^5^ cells/ml onto an eight‐well cell culture slide (Biologix, Shandong, China) and incubated with 1 μg/ml of wild‐type or mutated (R33W) ANG protein (recombinant proteins contain N‐terminal methionine, Novoprotein Scientific, Summit, NJ) for 30 min at 37°C. Cells were fixed with 4% paraformaldehyde (Sigma Aldrich Ltd., Budapest, Hungary) and permeabilized with 0.25% Triton X‐100 (Sigma Aldrich Ltd.). Nonspecific binding was prevented by incubation in 1% BSA and 1% normal goat serum (Sigma Aldrich Ltd.) followed by incubation with an anti‐ANG mouse monoclonal antibody [14,017.7] (Abcam, Cambridge, UK) overnight at 4°C and Alexa Fluor 647 anti‐mouse IgG (Sigma Aldrich Ltd.) for 90 min at room temperature. The nuclei were counterstained with 4′,6‐diamidino‐2‐phenylindole dihydrochloride (Sigma Aldrich Ltd.). Slides were mounted by Fluoromount (Emergo Europe, Hague, Netherlands) and visualized using a Zeiss LSM 880 Laser Scanning Microscope fitted with PMT and GaASP detectors (Carl Zeiss Microscopy GmbH, Jena, Germany). ImageJ software (Ver. 1.52) was used to determine mean signal intensity of each cell and the cytoplasm and nucleus of each cell in at least three fields of view per experiment and condition (Schneider, Rasband, & Eliceiri, 2012). The ratio of nuclear/whole staining intensity was calculated to determine nuclear localization intensity of ANG, two‐tailed *T*‐test was used to determine significance with a significance level set at *p* ≤ 0.05.

## 3 RESULTS

Mutation analysis of the *ANG* gene revealed three coding‐region mutations in four ALS patients (4/136 patients; 2.94%). One of the detected mutations affects the signal peptide (M‐24I) and the two are located in the mature ANG protein (R33W, V103I). These mutations were not detected in 112 age‐matched healthy controls.

*ANG* sequence analysis confirmed the presence of the rs11701 polymorphism in our ALS and control populations. We did not observe significant differences in allelic distributions between sALS patients, (minor allele frequency, MAF(G) = 0.118) and controls (MAF(G) = 0.171).

### 3.1 M‐24I mutation

The known M‐24I mutation (c.3G>T) was detected in two patients. One of the patients, who was described in our previous study (Tripolszki et al., 2017), also carried a mutation in the *SOD1* gene (V14M).

Clinical report: The other patient with the *ANG* M‐24I mutation was a 79‐year‐old woman. Her symptoms started 6 months before the examination with progressing dysphagia and dysarthria. On examination she was anarthric with increased gag and soft palate reflex and exhibited difficulty moving the tongue, which showed slight muscle atrophy. Deep tendon reflexes were increased in the extremities; however, she did not complain of weakness, even though signs of lower motor neuron damage were detected with electromyography (EMG). The revised ALS Functional Rating Scale (ALSFRS‐R) was 33 (the normal score is 48). The Mini‐Mental State Examination (MMSE) score was in the normal range: 27 (the maximum score is 30). The patient could communicate in writing. The initial symptoms indicated pseudobulbar palsy.

### 3.2 V103I mutation

A recurrent missense variant (c.379G>A; V103I) was identified in the mature ANG protein.

Clinical report: The patient with the V103I mutation was a 63‐year‐old man presenting with severe weakness of the right arm due to muscle atrophy. Slight bulbar and pseudobulbar weakness was noted, and the muscle strength also diminished in his left arm without severe atrophy of the muscles. The lower extremities were relatively strong. The patient could walk normally. Nevertheless, fasciculations were seen in all the muscles of the extremities and observed together with fibrillations and positive sharp waves on EMG examination, which are indicative of lower motor neuron lesions. The ALSFRS‐R was 41. The disease started 1.5 year before the examination. He was operated with coarctation of the aorta and an artificial valve was implanted 15 years before the onset of ALS. He was on continuous Coumadin treatment. The result of the MMSE was 28 and was comparable with the global impression that the patient had no intellectual deficit. The initial disease sign was lower motor neuron damage of the right arm.

### 3.3 R33W mutation

A missense variant (c.169C>T; R33W) located in the nuclear localization signal was identified in one patient (Figure 1). This variant has not been previously reported in an ALS patient, but occurs with a low allele frequency in the gnomAD database (MAF(T) = 0.00001624). Six of the seven computational variant‐effect prediction tools used predicted R33W to be pathogenic.

#### 3.3.1 Molecular dynamics simulations

MD simulation of the R33W variant protein was carried out to model structural and dynamics changes in the functional sites compared to the wild‐type protein. The backbone root mean square deviation (RMSD) of the R33W variant obtained from MD simulations showed that, although the fluctuations were higher than the wild‐type during the first 15 ns, it subsequently stabilized and converged as observed in the wild‐type ANG (Figure S1). This suggested that the Arg33 to Trp33 mutation may not affect the structural stability of the mutant.

#### 3.3.2 Conformational switching of catalytic residue His114 causes loss of ribonucleolytic function in the R33W protein

**Figure 2:**
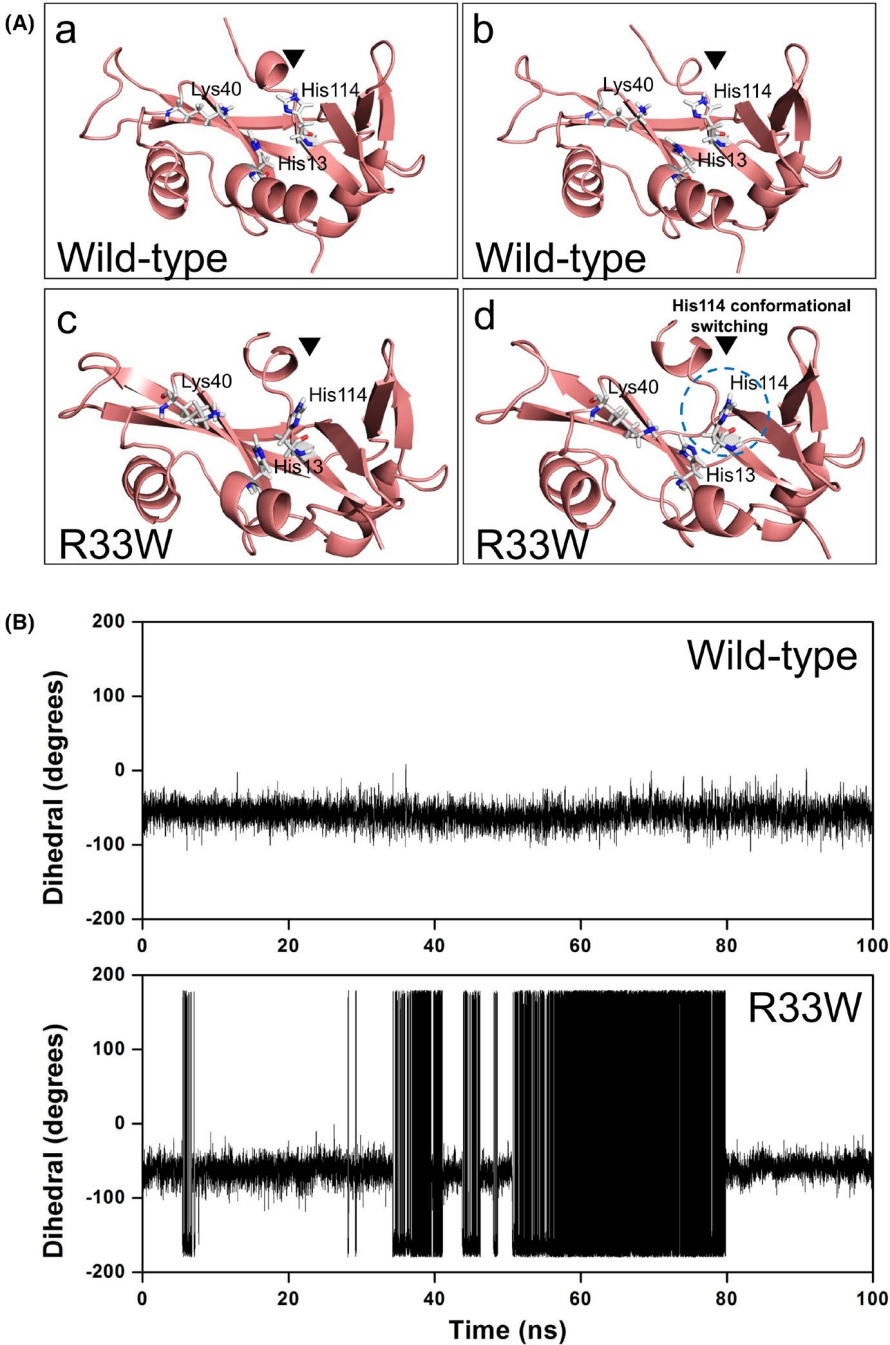
Conformational switching of catalytic residue His114 in R33W mutant. (A) Snapshots extracted from molecular dynamics (MD) simulation of wild‐type angiogenin (ANG) (a and b) showing the native and stable conformation of catalytic residue His114 as compared to the conformational altered His114 switching in R33W mutant (c and d) as observed during the MD simulation. (B) Change in HA‐CA‐CB‐CG dihedral angle of catalytic residue His114 during MD simulation as a function of time. Catalytic residue His114 changed its conformation from native −80º during the simulations, indicating its role in probable loss of ribonucleolytic activity, whereas wild‐type ANG maintained a stable His114 conformation

MD simulations were carried out to examine whether the R33W variant exhibits a loss of ribonucleolytic activity. A 100 ns all‐atom simulation of the R33W variant protein revealed that one of the catalytic residues, His114, exhibited a characteristic conformational switching from its native position during the simulations (Figure 2A). As established in our previous studies (Padhi, Jayaram, & Gomes, 2013; Padhi, Vasaikar, Jayaram, & Gomes, 2013; Padhi et al., 2014), this conformational switching of His114 results in loss of ribonucleolytic activity. The R33W variant showed a similar behavior, where the quantitative HA‐CA‐CB‐CG dihedral angle measurement of His114 deviated from the mean −80° position to −179° (Figure 2B). Thus, based on the MD simulations of the wild‐type and R33W variant, we predict that the R33W variant is possibly reduces ribonucleolytic activity.

**Figure 3:**
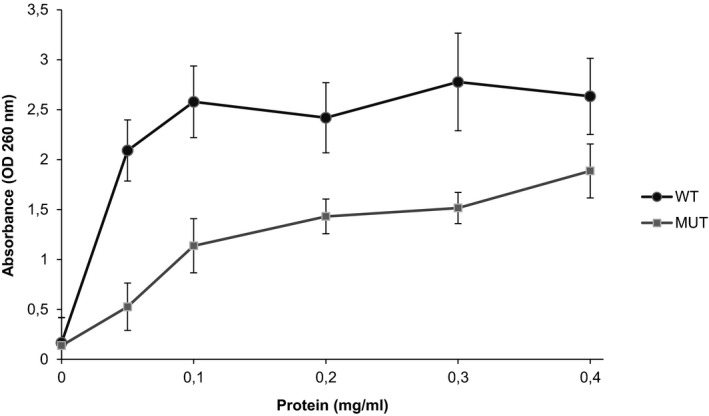
Ribonucleolytic assay of wild‐type (WT) and mutant (MUT) R33W angiogenin (ANG) proteins. Enzyme activity was measured using yeast tRNA as substrate. Increasing concentrations (0.05–0.4 mg/ml) of WT and R33W (MUT) proteins were incubated with yeast tRNA (2 mg/ml) at 37°C for 2 hr. Undigested tRNA was precipitated with perchloric acid. The absorbance of the supernatants was measured at 260 nm. Data were collected from three independent measurements. Relative enzyme activity of the MUT ANG was calculated as compared with the WT protein (100%). The activity differences at concentrations 0.1, 0.2, 0.3, and 0.4 mg/ml were calculated

To validate the simulation‐based prediction, we performed the ribonucleolytic assay using yeast tRNA as substrate and our quantitative analysis showed that the R33W mutant retained only 57.4% of the ribonucleolytic activity compared to wild‐type ANG (considered 100%) (Figure 3). The result of this enzymatic activity assay was consistent with the MD simulation results.

#### 3.3.3 Local folding of nuclear localization signal abolishes nuclear translocation activity of the R33W variant

**Figure 4:**
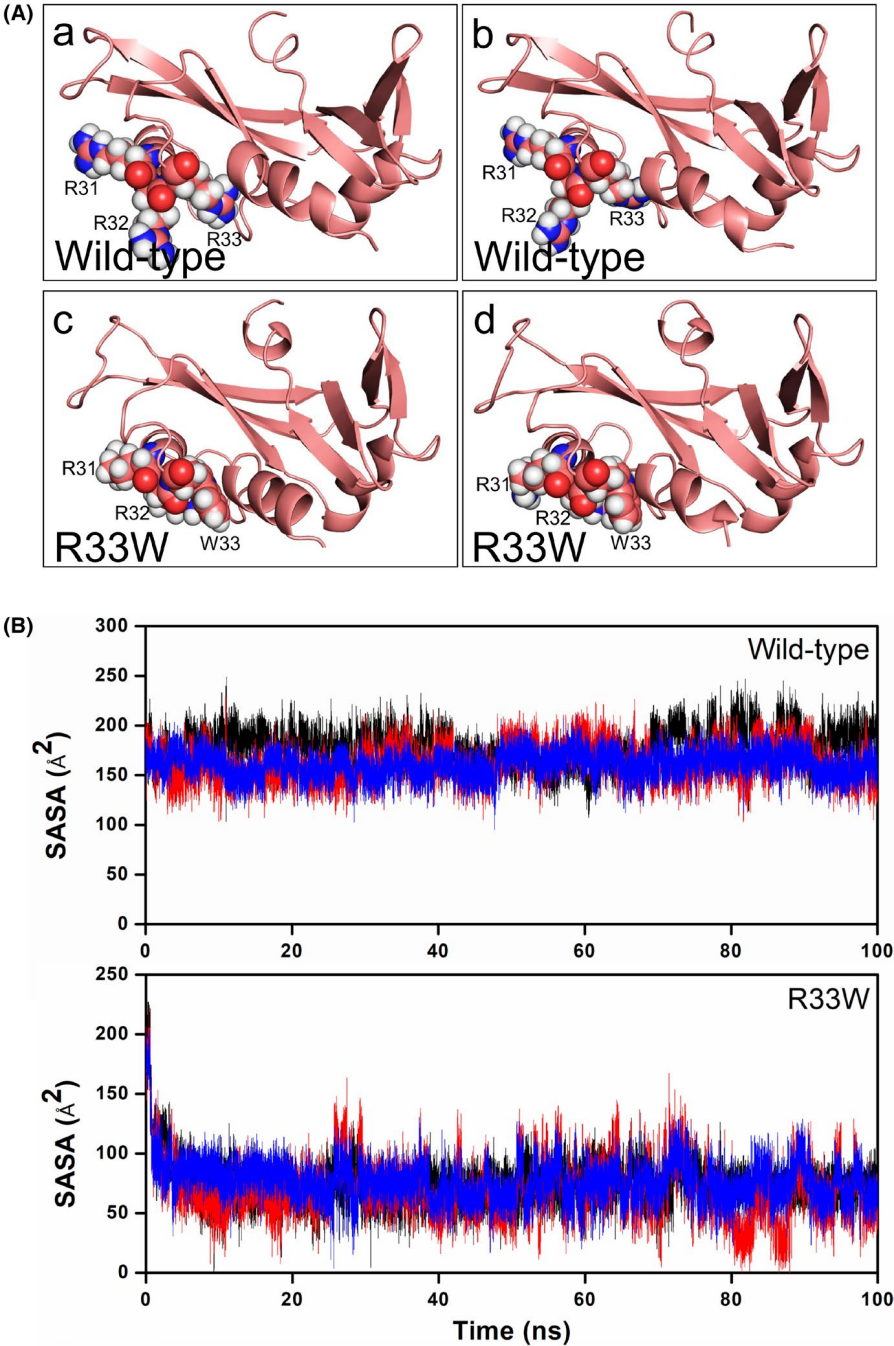
Local folding of nuclear localization signal residues in R33W mutant. (A) Snapshots extracted from molecular dynamics (MD) simulation of wild‐type angiogenin (ANG) (a and b) showing its open conformation and loose packing of ^31^RRR^33^ nuclear localization signal residues as compared to the closed and tight packing of ^31^RRW^33^ residues (c and d) observed during MD simulations. (B) Computed solvent‐accessible surface area (SASA) from MD simulations of nuclear localization signal residues ^31^RRR^33^ or ^31^RRW^33^ for wild‐type ANG and R33W mutant, respectively. Reduced SASA in R33W mutant indicates that it may lose its nuclear translocation ability as compared to wild‐type. SASA of R31, R32, and R33/W33 are shown in black, red, and blue, respectively

**Figure 5:**
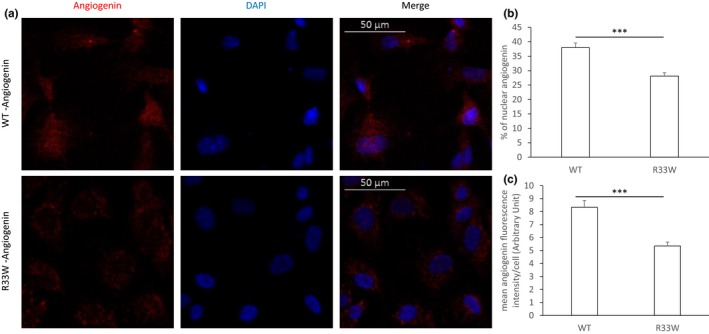
Nuclear translocation of wild‐type (WT) and mutant angiogenin (ANG) in human umbilical vein endothelial cells (HUVEC) cells. HUVEC cells were incubated with 1 μg/ml of WT ANG or mutant ANG for 30 min and subsequently immunostained for ANG. Nuclei were stained with 4′,6‐diamidino‐2‐phenylindole (DAPI). Images show the representative result of four experiments. (a) WT ANG accumulates both in the cytoplasm and nucleus of the HUVEC cells, whereas mutant (R33W) ANG accumulated predominantly in the cytoplasm of the cells. Individual channels as well as merged images are shown. (b) Nuclear translocation was quantified by measuring mean signal intensity levels of ANG staining in nucleus and cytoplasm of cells for at least three fields of views. Quantification of nuclear translocation of WT and mutant (R33W) ANG showed a significant decrease in nuclear staining in the R33W‐treated samples (two‐tailed Student's *t*‐test, *p* < 0.0001). (c) Mean cellular signal intensity levels of ANG was determined for at least three fields of views, which showed a significant difference between the intensity of staining of the WT and mutant protein (two‐tailed Student's *t*‐test, *p* < 0.0001), suggesting a difference in the cellular internalization of the protein

The nuclear translocation activity of the R33W variant was investigated from MD simulations. Our 100 ns simulation of R33W variant showed that the key nuclear localization signal residues 31RRW33 undergo local folding and becomes less accessible to solvent as compared to wild‐type (Figure 4A). This resulted in a reduced SASA in the R33W variant compared to the wild‐type, which retained an open conformation and loosely packed 31RRR33 residues (Figure 4B). Since the 31RRR33 residues are known to play a pivotal role in the nuclear translocation of ANG, we predicted that a decrease in SASA due to the local folding and close packing of 31RRR33 residues originated due to the replacement of hydrophilic Arg33 to hydrophobic Trp33 would probably lead to the loss of nuclear translocation activity in R33W variant. Our MD simulated results were verified through nuclear translocation assay performed on cultured HUVEC cells. Treatment with wild‐type and mutant R33W protein and subsequent immunofluorescent staining demonstrated that while wild‐type ANG translocated into the nucleus, the R33W mutant remained primarily in the cytoplasm (Figure 5a, for isotype staining see Figure S3). Cells with nuclear or cytoplasmic staining were counted and while wild‐type ANG showed nuclear/cytoplasmic staining in 24/34 cells per field of view, the R33W mutant showed nuclear/cytoplasmic staining in 14/37 cells per field of view on average (*p* = 0.001, determined by chi‐square test, the chi‐square statistic is 10.6984). Decreased translocation of R33W ANG into the nucleus was also confirmed by quantification of nuclear/signal intensity (Figure 5b). Besides the difference in nuclear translocation activity, whole cellular staining intensity was decreased in the R33W ANG‐treated samples compared to the WT ANG‐treated samples (Figure 5c), suggesting a defect in the cellular recognition process and internalization of the mutant protein. Untreated HUVEC cells showed no staining for ANG (Figure S3a).

Clinical report: The R33W variant was carried by a 55‐year‐old male patient presented to our clinic with severe distally dominant atrophy of the muscles in the arms with weakness. He noticed the initial symptoms 1 year before examination. A gradually worsening difficulty of swallowing developed about half year after the initial symptoms. The tongue was atrophic and the articulation was compromised. He lost 25 kg weight since symptom onset. On physical and on EMG examinations, frequent fasciculations and fibrillations were observed in all examined myotomes, even in the lower extremities. The gag‐ and soft palate reflexes were absent, and no deep tendon reflexes in the upper extremities could be elicited. However, reflexes were exaggerated in the lower extremities. The patient could walk well but his arms were useless. He had been treated for depression in the previous 2 years. The ALSFRS‐R was 35. The MMSE could not be performed because of the patient's compromised speech and severe weakness of the hands. Nevertheless, there were no signs of mental deterioration.

## 4 DISCUSSION

In this study, we identified three mutations in the coding region of the *ANG* gene, one of which has not been reported previously in ALS patients. The frequency of *ANG* mutations in our Hungarian sALS cohort (4/136; 2.9%) is higher than previously reported. According to previous studies, the frequency of *ANG* mutations in patients with no known family history is 0.7%, with higher frequencies in some populations (e.g., 1.4% and 1.9% in patients of Irish or Scottish origin, respectively, Greenway et al., 2004, 2006) and 2.4% in patients with positive family history (van Es et al., 2011).

### 4.1 M‐24I mutation

The M‐24I mutation was first described by Conforti et al. (2008) and was detected in two Hungarian patients in this study. The biological function of the signal peptide is not entirely understood; hence it is difficult to predict the effect of the M‐24I mutation. Nonetheless, it affects the start codon (ATG) of the gene, which may influence the correct translation of the protein.

One of the patients with the *ANG* M‐24I also carried a mutation in the *SOD1* gene (V14M) and was described in our previous study (Tripolszki et al., 2017). The co‐occurrence of different variants in ALS‐associated genes was also detected in other ALS cohort screenings (Kenna et al., 2013; Krüger et al., 2016).

### 4.2 V103I mutation

The V103I mutation affects an amino acid that is conserved in mammals and it was first detected in an ALS cohort of Chinese origin (Zou et al., 2011). According to previously reported molecular simulation results of the V103I variant structure, a hydrogen bond interaction network connecting from I103 to H114 was proposed (Padhi et al., 2012). Enzyme activity assays showed that the V103I mutant retains 54.1% of enzymatic activity toward tRNA (Bradshaw et al., 2017). Observing SASA values of the V103I mutant, it is predicted that the nuclear translocation activity is also impaired (Padhi et al., 2012).

### 4.3 R33W mutation

The R33W missense variant, located in the nuclear translocation signal of the mature protein, has not been identified in ALS patients before. To understand the role of the mutation in disease manifestation, a computational and functional analysis of the R33W mutant was performed. Our MD simulations showed that the R33W mutation results in partial loss of ribonucleolytic activity and impaired nuclear translocation activity. The ribonucleolytic assay and nuclear translocation assay of the R33W ANG protein confirmed the MD results. According to the ribonucleolytic activity assay, the R33W mutant protein retained 57.4% of catalytic activity of the wild‐type protein. Previous studies described that the reported disease‐causing *ANG* mutations—except variant R121C—show a reduced level of catalytic activity in a range of 0%–95% compared with the wild‐type ANG (Bradshaw et al., 2017; Padhi et al., 2014; Wu et al., 2007).

Since the R33W variant affects the key amino acid of the nuclear localization signal of ANG, this function of the mutant protein is also compromised. Our nuclear translocation assay showed that the alteration of Arg33 to Trp33 in ANG significantly reduced its entry into the nucleus. Amino acids ^29^IMRRRGL^35^ are part of the nuclear localization signal and are involved in nuclear translocation of ANG. The arginine residues ^31^RRR^33^ are critical in governing the nuclear translocation of ANG: R33 is essential and residues ^31^RR^32^ modulate the process. Moroianu and Riordan (1994) described that the R33A mutant ANG protein is not translocated to the nucleus and lacks angiogenic activity (Moroianu & Riordan, 1994). Previous studies showed that other ALS‐associated *ANG* mutations (S28N, L35P, P112L, K17I, K17E, Q12L) also result in partial or complete loss of nuclear translocation capability due to the changes in the folding of the ^31^RRR^33^ residues and subsequent reduction in SASA (Bradshaw et al., 2017; Padhi et al., 2014; Thiyagarajan, Ferguson, Subramanian, & Acharya, 2012; Wu et al., 2007).

Mature ANG contains three distinct functional sites: a receptor binding region, a catalytic triad responsible, which is for ribonuclease activity, and the nuclear localization signal. All three functional regions are needed for its angiogenic activity (Moroianu & Riordan, 1994). Alterations in the structure of ANG and dynamics result in loss of ribonucleolytic activity and/or nuclear translocation ability, which may lead to ALS.

For the R33W variant, a strong correlation between the computational simulation predictions and functional analyses was observed. The evidence from clinical, computational, and functional analyses support the deleterious effect of the R33W variant detected in this study. Our findings also confirm the relevance of *ANG* mutations in ALS and points out the variability among populations of different ethnic origin. It underscores a substantial role of *ANG* variants in Hungarian ALS patients, and suggests the importance of further large cohort studies.

## CONFLICT OF INTEREST

The authors report no conflict of interest regarding this work.

## Data Availability

The data that support the findings of this study are available from the corresponding author upon reasonable request.
