# Supplementary figures and images for: Angiogenin mutations in Hungarian patients with amyotrophic lateral sclerosis: Clinical, genetic, computational, and functional analyses
Source: Brain Behav. 2019 Apr 25;9(6):e01293. doi: 10.1002/brb3.1293 (PMC6576160; doi:10.1002/brb3.1293)

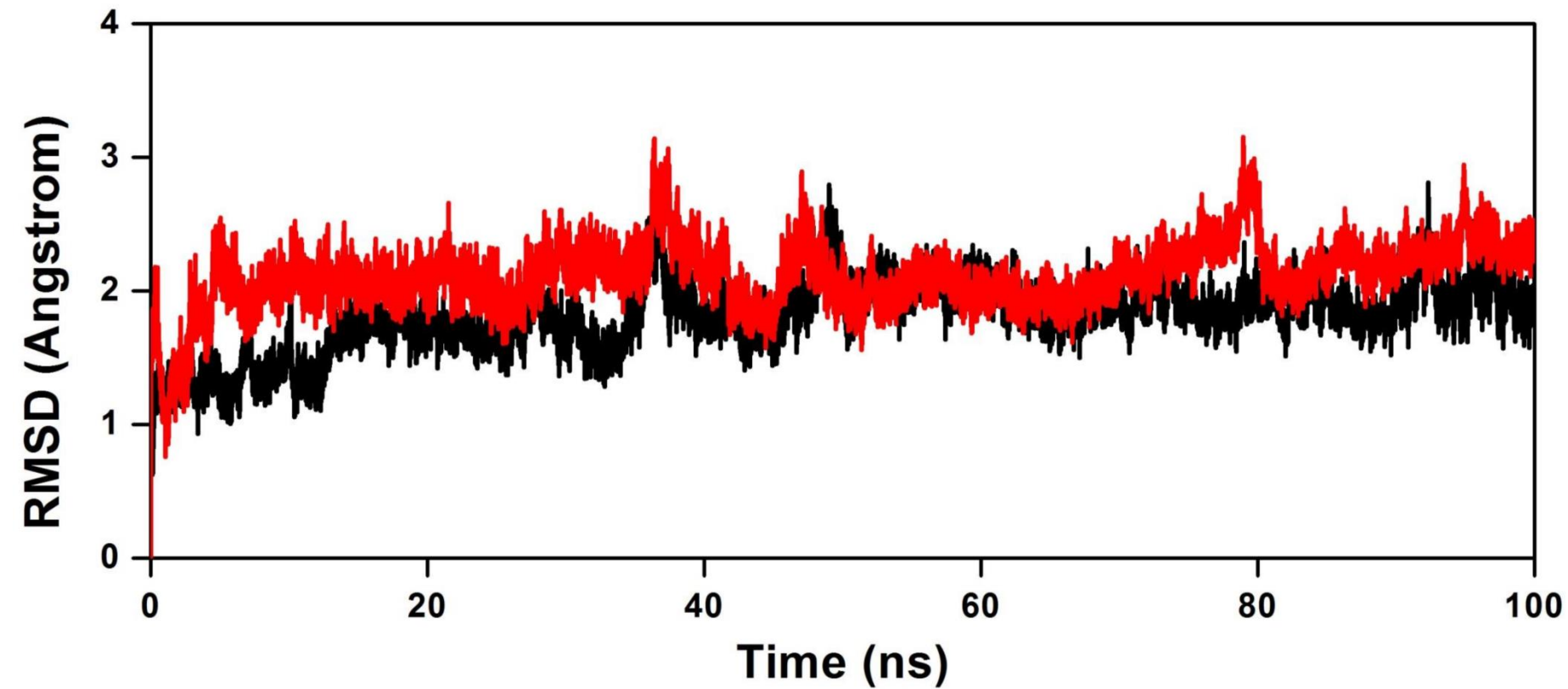

Supplement: Supplementary file 1 [file BRB3-9-e01293-s001.pdf]

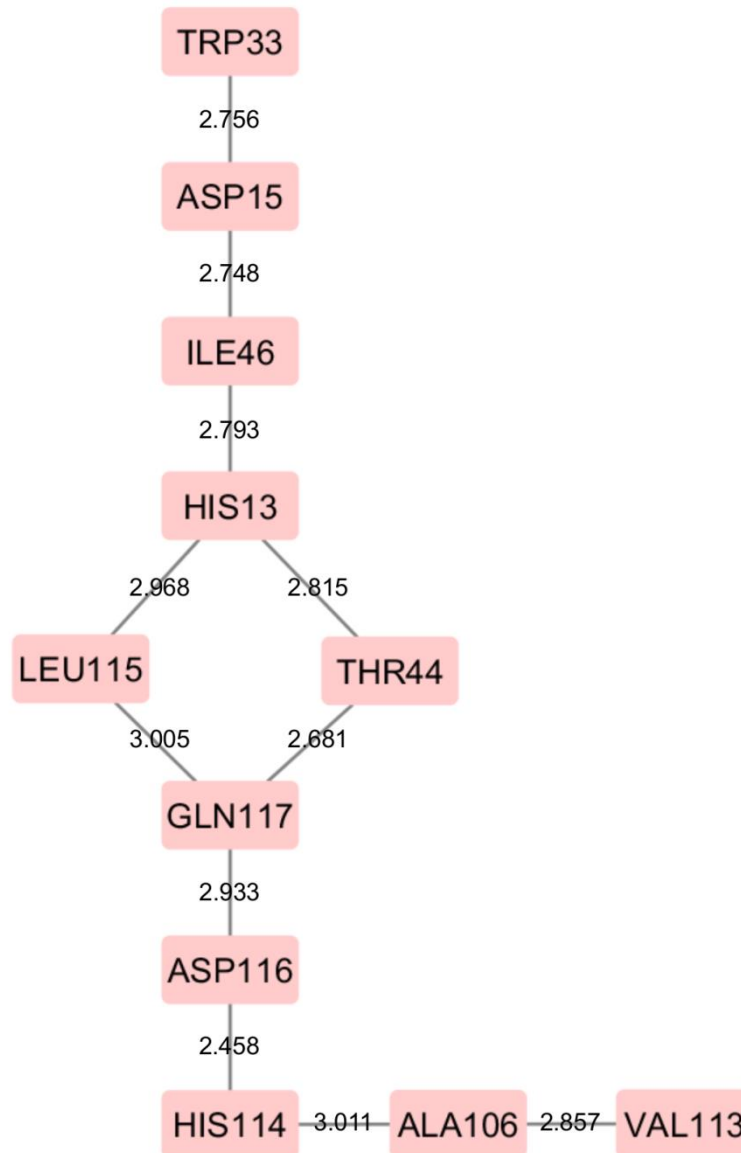

Supplement: Supplementary file 2 [file BRB3-9-e01293-s002.pdf]

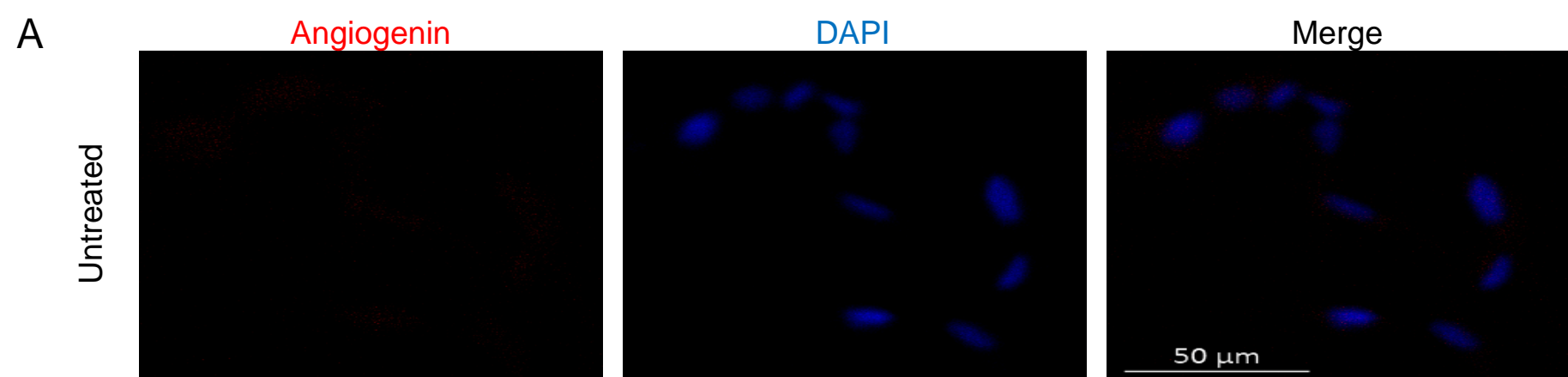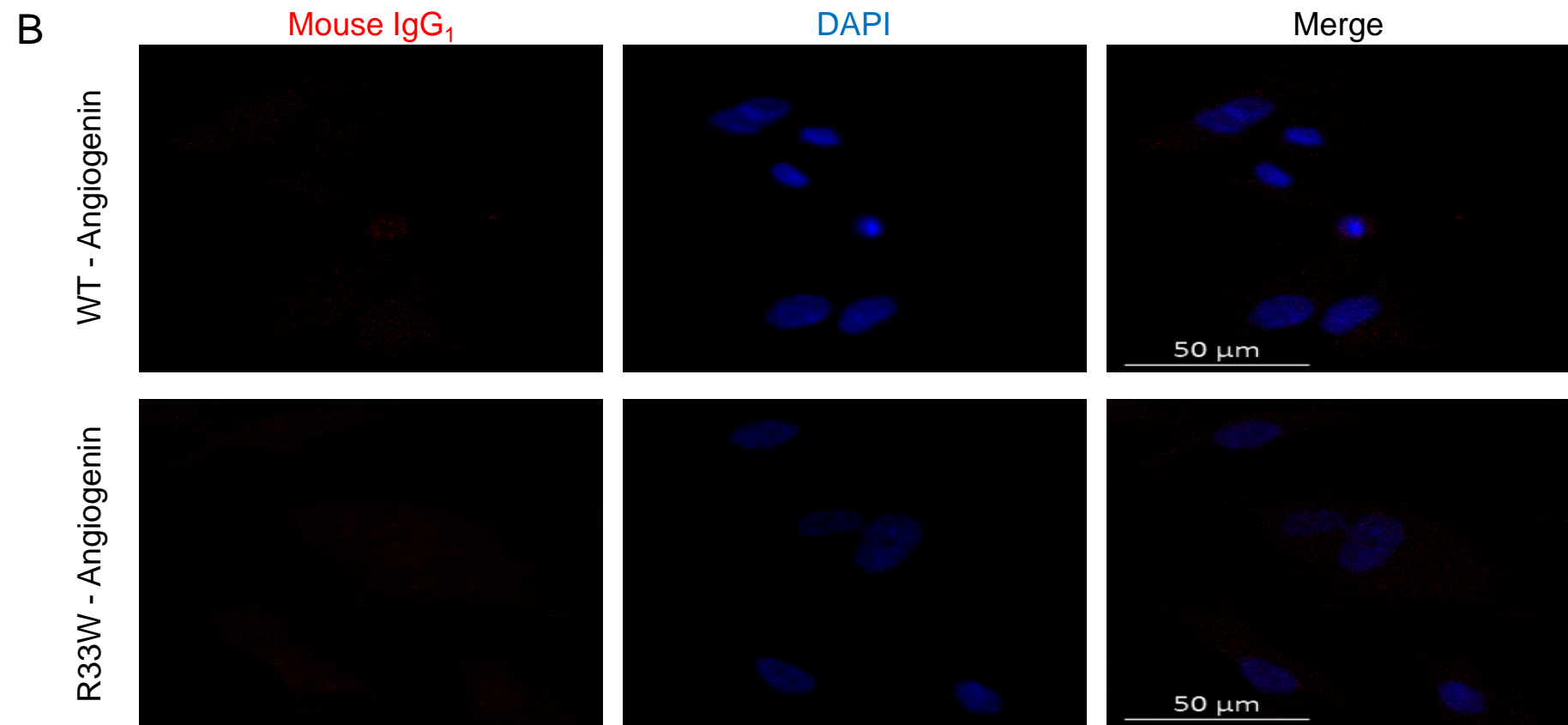

Supplement: Supplementary file 3 [file BRB3-9-e01293-s003.pdf]
